# The influence of the brand image of green agriculture products on China’s consumption intention——The mediating role of perceived value

**DOI:** 10.1371/journal.pone.0292633

**Published:** 2023-10-05

**Authors:** Hui Yang, Pengpeng Zhang, Haijiao Liu

**Affiliations:** 1 College of Economics and Management, Northeast Agricultural University, Harbin, China; 2 Modern Agricultural Development Research Center, Northeast Agricultural University, Harbin, China; Xuzhou University of Technology, CHINA

## Abstract

Green agriculture can minimize the negative impact of agriculture on the environment. As countries around the world strongly advocate green production and green life style, not only is consumers’ awareness about green consumption rising, but the demand for green agricultural products at home and abroad is gradually increasing as well. Brand image has been a crucial factor for consumers to make their final purchasing decisions, thus playing a critical role in influencing the purchasing decisions of consumers. Based on the theory of the brand image, this paper undergoes a comprehensive theoretical and positive analysis and explores the influence mechanism of brand image of green agricultural products on consumers’ purchasing intention. A hypothetical model with perceived value as mediators is constructed by us to examine the influence of brand image of green agricultural products on consumers’ purchasing intention. A quantitative study was conducted for a random sample of 341 consumers who purchased green agricultural products in China according to a questionnaire-based survey using a cluster random sampling technique. The study showed that the overall image of agribusiness, the image of agricultural products, the social image of agribusiness, and the image of consumers are all positively related to consumption intention. The overall image of agribusiness, the image of agricultural products, the social image of agribusiness, and the image of consumers have a positive influence on perceived value. Moreover, perceived value plays a part in the mediating role in the influence of the overall image of agribusiness, the image of agricultural products, the social image of agribusiness, and the image of consumers on consumption intention. These findings shed lights on enterprises in establishing a scientific and effective brand strategy and building an excellent brand image. The research conclusion can provide new insight into how to enhance the consumption willingness for green agricultural products and promote sustainable development.

## Introduction

The development of green agriculture is an effective way to achieve sustainable agricultural development. It is important to ensure food security, improve the supply capacity of agricultural products, promote healthy land development, and achieve green development in all countries [1]. Economic activities resulting from rapid economic growth can cause serious environmental damage to the planet [2]. The environment in which agricultural products are grown is subject to severe resource and environmental constraints due to climate change, air pollution, waste generation, and natural disasters, which pose a serious threat to sustainable agricultural development. This affects not only the organisms but also the economic and social status of people [3]. The consumer movement is calling on governments and businesses to take greater action to address climate change, which has led to a shift in consumer preferences. Internal and international pressures have also increased the pressure on governments and businesses in various countries to adjust their economic practices to reduce or offset the impact of various activities on the environment [4]. The spread of COVID-19 drives a huge surge in demand for agricultural products, however, the challenges surrounding the production, service, logistics and labor availability of agricultural products have posed a constant threat [5]. Aiming to empirically evaluate the impact of green capabilities on green purchasing practices, research scholars made a study of the structural equation modeling methods based on the covariance, thereby boosting the triple-bottom-line performance of manufacturing organizations. The results indicate a positive correlation between green capabilities and purchasing habits [6]. To a certain extent, green product consumption can reduce the impact on the environment. The so-called green products are those that are produced in accordance with the principles of sustainable development, can be safely purchased, and are of good quality. In recent years, there has been a significant increase in the demand for green products on a global scale. Consequently, the production of green products has also increased significantly [7]. Maintaining ecological balance and reducing environmental damage are important prerequisites for human survival and social development. And the quality and safety of agricultural products are closely related to human health and life safety [8]. The development of green agriculture can increase farmers’ income, reduce agricultural pollution, and solve the problem of food safety [9]. In a market economy, the main incentive for producers to develop and promote green agriculture products depends on the willingness of consumers to buy green agriculture products [10]. With the rapid advancement of production technology, the competition in the agricultural products market has become more and more intense. This fierce competition manifested in the green agriculture products market is not only the competition between prices but also the competition between quality, popularity, and reputation. The brand image can be regarded as a key factor indicating how consumers feel about a brand and whether there is a positive relationship between the brand and the consumer. Business managers can measure brand images, identify brand images that can serve as positive associations, and focus on building those images in their brand management process. Given this, the ability of business managers to understand how consumers perceive brands is critical to successful brand management [11]. For companies, implementing a branding strategy has become an important step to win and improve the competitiveness of the brand in the market. Enterprises need to think from the perspective of consumers to create a good brand image, which is also an important basis for consumers to choose the product. Therefore, it is important to help enterprises develop a good brand image for green agriculture products in order to improve green agriculture products consumption intention, promote green consumption, and achieve sustainable development as soon as possible.

A brand can be viewed as a promise to a customer about benefits they will receive from the companies’ product [12]. Brand image is a reflection of consumers’ perceptions of a brand, and consumers tend to remember brands with good brand images more easily, thus increasing their loyalty to the brand. Without any other prior knowledge of the product, consumers will use the brand image to help them evaluate their purchase decision [13]. A green brand image can create a competitive advantage [14]. The contributions of Aaker and Keller to brand management have provided new impetus for future in-depth research in this area [15, 16]. Aaker defined brand image as a set of associations that are usually organized in a meaningful way. The brand association can take any form that can be associated with brand memory, such as product attributes, customer benefits, or relative price. Contrary to Aaker’s view, Keller believed that brand image is the consumer’s perception of a brand that reflects its meaning and is preserved in memory in the form of a network of associations [17]. Biel argued that brand image is a subjective consumer response to a brand, which is a collection of attributes and related associations that consumers associate with a brand in their minds [18]. Currently, in addition to the study on theories of brand image, many researches on brand image has also been conducted by empirically examining the relationship between brand image and different marketing structures and accordingly concluding that brand image exerts a positive impact on brand trust, customer satisfaction, and brand equity [19–21]. Some researches manage to evaluate the brand image effect of the corporate combination of the two or more companies from the perspective of marketing, concluding that the greater the perceptive difference between the brands of the acquiring firms, the more brand equity the acquirer will reap [22]. A brand image with environmental benefits is personified (cute and cool). As brand trust plays the mediating role in self-serving advertising, the marvellous image of the brand is more likely to increase the willingness to consume green products [23].

Green agriculture products have a special role in environmental challenges and sustainable development. However, consumer awareness of green agriculture products is low, which leads to low consumption intention [24]. As regards the reason why some agricultural retailers have less willingness to spend, a study conducts a questionnaire survey on 620 samples in China, aiming to identify the drivers of omni-channel consumer purchasing intention in agricultural products retail. The results show that single-channel shopping cost, reference groups, positive online reviews, and single-channel perceived risk have a significant positive impact on the omni-channel purchase intention of agricultural products, and perceived value plays a mediating role [25]. When green agriculture products companies have a good brand image, to a certain extent, it can enhance consumer awareness and thus increase consumer willingness. Consumer perception value is important as a trade-off between the cost consumers pay for a product or service and the benefits they perceive for that product or service [26], so the customer-perceived value should also be an important factor influencing brand image on consumption intention. In recent years, customer perceived value theory has been gradually applied to the field of agricultural economics to analyze the factors influencing farmers’ intentions and behaviors [27], which suggests a logical relationship between green agriculture product brand image, customer perceived value and consumption intention.

In order to create a more significant value space, green agricultural development should be quickly transferred to brand image shaping, perceived value, and increase consumer willingness. Therefore, this paper explores the influencing mechanism of green agricultural products brand image value creation on consumption intention based on brand image theory and SOR model, on this basis, the structural equation model is used to test the research hypothesis empirically to provide quantitative scientific data for green agricultural products enterprises to conduct the brand image value creation and marketing strategies. The question this paper seeks to answer is the following questions: To what extent does the composition dimension of green agricultural products brand image affect the consumption willingness of green agricultural products? To what extent does the composition dimension of green agricultural products brand image affect the perceived value of Chinese consumers? Does the perceived value play an intermediary role between the green agricultural products brand image and the willingness to consume?

In terms of its main contribution, although many scholars have emphasized the importance of the sustainable development of agriculture, green production, and improvement of the purchasing intention of agricultural products and other related issues, there are few articles conducting systematic research from the perspective of brand image and perceived value of green agricultural products. By reading a large amount of literature in related fields at home and abroad, this paper classified the constituent dimensions of the brand image of green agriculture products. Using customer perceptive value as a mediating variable, this paper constructs a theoretical model about how brand image of green agricultural product affect consumption intention, exploring the impact of different dimensions of brand image of green agricultural product on consumption willingness and the role of perceptive value between the two. This will provide theoretical contributions to the literature such as green agriculture and brand image. As the country and the government pay more and more attention to green consumption, the research conclusion of this study can also provide scientific and effective marketing strategies for the development of green agricultural products enterprises, thus improving the consumption willingness of green agricultural products, and boosting the comprehensive and sustainable development.

## Theoretical basis and research hypothesis

### Theoretical basis

#### Brand image theory

In the 1950s, American industrial enterprises grew rapidly. In this era, which was accompanied by an increasing abundance of products, competition between companies’ products grew. At this time, in order to create a difference between the company’s products and its competitors’ products, each company has to find a unique selling point for its own products so as to achieve differentiated marketing [28]. It was in this historical context that Rosser Reeves proposed the Unique Selling Proposition (USP) [29]. Due to the rapid development of social productivity and economic prosperity, more and more enterprises are producing homogeneous products. With the maturity of the buyer’s market, the business philosophy of enterprises has gradually changed from the old sales idea to the marketing approach. In this context, David Ogilvy defined brand image as the public, and consumers will evaluate the brand based on the information they have learned. In this way, a perception of the brand is formed, and the brand image is what exists in the minds of the public and consumers [30]. For advertising, it must be shifted from the original appeal to the unique function of the product to the shaping of the brand image, which ultimately determines the overall image of the brand market positioning. Long-term and lasting external stimulation from advertising creates an overall impression and perception of a brand. Researchers generally believe that brand image is an important factor in influencing consumers’ purchasing decisions. A reputable brand image is an important aspect of helping customers make purchase decisions effectively and satisfactorily, and it will ultimately provide good results and associations in the consumer’s long-term mind [31]. In brand promotion, consumers will often enhance their perception of the product because of the good corporate image of the company carrying out the promotion, the high-cost performance, high quality, the high nutritional value of green agricultural products, or the excellent and innovative product packaging, etc., and then generate consumption intention.

#### SOR model

The name of the SOR Model is composed of the initials of the three variables Stimulus, Organism, and Response. "S" denotes external stimuli (both micro and macro stimuli); "O" denotes the organism, including the consumer perceived value (customer perceived value); and "R" denotes the response variable (i.e., the consumer’s inner drive to respond to various stimuli from the external environment) [32]. The model stated that consumers make a complete purchase decision because they are motivated by internal and external stimuli. Then they are motivated to make a purchase, i.e., they are motivated by the psychological state of the internal and external stimuli, and then they make the corresponding behavior and willingness [33]. Stimuli are external forces that affect the individual’s mental state, the organism is the internal processes and structures, and the response is the final outcome of the individual’s behavior, which may be positive or negative [34]. Many scholars have applied this model to the study of Consumer behaviour, which shows that this model can effectively predict consumers’ purchase intention [35–37]. Some scholars have built the influence mechanism and research model of brand extension evaluation from the perspective of user perception based on SOR model. The results show that brand image and perceived fit have positive effects on extension evaluation, and perceived fit plays an intermediary role in the influence of brand image on extension evaluation [38].

Based on the SOR Model and Brand image theory, this paper explores whether green agriculture product brand image plays a role in consumption intention, introduces customer perceived value, and constructs a mediation path of green agriculture product brand image on consumption intention. Based on the SOR model and brand image theory, this paper explores the mechanism of green agriculture product brand image on consumption intention and how it works.

### Research hypotheses

#### Green agriculture product brand image metrics

David Ogilvy, an American advertising guru, defined brand image as the reflection in the consumer’s mind of the perceived existence of a brand as a result of a series of associations made by the consumer [39]. Regarding how to classify the dimensions of brand image from different perspectives, domestic and foreign scholars in related fields have established different measurement models. Biel’s brand image model consists of three dimensions: corporate image, product or service image, and user image. In addition, Biel also divided the sub-dimensions into deeper levels according to the "hard" and "soft" attributes of each dimension. In Biel’s view, the hard attributes provided by a product or service are tangible or functionally specific perceptions; user image and company image provide soft or emotional attributes, such as excitement, trust, and fun [18]. Keller applied the brand association network model to classify the brand image into three association categories, namely attributes, benefits, and attitudes. Furthermore, Keller further refined attributes and benefits into product-related attributes and non-product-related attributes [16]. Based on the brand image, Aaker proposed the ten elements model of brand assets, which is measured from five aspects: brand loyalty, perceived quality, brand association, brand awareness and other exclusive brand assets [40]. Krishnan believes that the brand image should be analyzed from the four aspects of the brand association (quantity, uniqueness, preference, and formation source). Krishnan’s model considers the brand image from the perspective of brand association [41]. Fan Xiucheng proposed a four-dimensional model of the brand image from the perspective of the brand identity system based on his own research characteristics by referring to the mature brand image models in foreign countries. The model includes a corporate dimension, a human dimension, a product dimension, and a symbolic dimension [42].

This study proposed a measurement system consisting of 4 dimensions and 12 indicators for green agriculture product brand image, specifically, including the image of the agribusiness, the image of the agricultural products, the social image of the agribusiness, and the image of the consumers by combing the literature and considering the brand image measurement model proposed by previous authors, and taking Chinese consumers as the research object. The corporate image of the agribusiness refers to the enterprise image which reminds consumers of various information related to the enterprise, and particularly the consumers’ usage experience and the emotional experience in using the enterprise products. The image of agricultural products refers to the perceptual cognition that the products bring to consumers, corresponding to the brand attributes that align with the functional or beneficial characteristics of the products or services themselves. Consumer image refers to the demographic characteristics of brand users, but also includes the user’s own personality, values and lifestyle [18]. The social image of the agribusiness refers to the image presented by the enterprise in the society, such as: frequent participation in public welfare activities, frequent benefits and less negative reports.

#### Relationship between green agriculture product brand image and consumption intention

By examining the direct relationship between brand image and consumers’ purchase intention, both domestic and foreign scholars have concluded that brand image is an important factor in consumers’ consumption intention for a product. Consumers are always susceptible to the influence of the concretized image. Therefore, efforts to create a good brand image of green agriculture products and enhance the recognition of brand image are conducive to increasing the consumption intention of green agriculture products. As the overall perception of a brand, brand image has an impact on consumers’ purchase intention [42]. Guan Hui and Dong divided brand image into three dimensions: brand performance, brand personality, and company image, and constructed a theoretical model to test that brand image influences empirically and determines people’s purchase and consumption intention [43]. From the perspective of green consumption, Li Hui’s study demonstrated that green brand image has a significant direct impact on customers’ consumption intention [44]. Sun Rui et al. proposed a six dimensional model of agricultural product brand image. The results indicate that the overall image of agribusiness, the overall image of agricultural products, the image of agribusiness employees and farmers, the scale image of agribusiness and farmers, and the social image and social service image of agribusiness and farmers all have a positive impact on the willingness to consume [45]. Wen Lina took Laoshan green tea as the research object and constructed a three-dimensional brand image model consisting of the image of the tea forest location, tea image, and tea drinker image. Through empirical analysis, the results showed that the image of the tea forest location, tea image, and tea drinker image have a positive correlation with consumers’ purchasing intention [46]. This shows that brand image plays a role in promoting consumption intention. A good brand image helps to enhance consumers’ willingness to buy. As a product of green agriculture, green agriculture products have been gaining more and more attention for their good ecological and economic benefits. In recent years, branding has also been intensified. In this regard, the following hypotheses are proposed in this paper:

**Hypothesis H1a:** The agribusiness image of green agriculture product brand has a positive impact on consumption intention.
**Hypothesis H1b:** The image of green agriculture products branded agricultural products has a positive impact on consumption intention.
**Hypothesis H1c:** The social image of agribusiness of green agriculture products brand has a positive impact on consumption intention.
**Hypothesis H1d:** The consumer image of green agriculture products brand has a positive impact on consumption intention.

#### Relationship between green agriculture product brand image and perceived value

The key for companies to survive in the traditional agricultural market is to create customer-perceived value for consumers to enhance consumption intention. Improving brand image through brand management is to help companies create customer-perceived value for consumers more effectively. Aaker pointed out that customer-perceived value is not only the consumer’s perception and evaluation of overall preferences for the consumption experience from a perceptual perspective but also the consumer’s perception and evaluation of benefits for the purpose of consumption from a utility perspective [15]. Brand image and service brand awareness have very different effects on the value and risk that consumers perceive in food products. Where brand image negatively affects perceived physical risk and positively affects brand preference [47]. In terms of the overall image, if a tourist destination or product has a good image, it can have a significant positive impact on the customer’s perceived value of the tourist or consumer [48]. Wiederhold and Martinez conducted an exploratory study to understand what factors influence the purchase of sustainable fashion products. Their study found that in addition to price, product availability, brand image, and consumption habits play a significant role in generating an attitude [49]. The consumer perceived value of a product is determined by attributes such as quality and health [50, 51]. Liu Chang explored the key constraints affecting customer perceived value by taking high-end consumer goods as the research object and found that product brand image has a significant positive impact on customer perceived value [52]. When the product is a well-known brand, customer perceived value is less sensitive to changes in product attributes and additional services. The sensitivity of customer perceived value to changes in product attributes and additional services is stronger when the product is less well-known [53]. Hence, when consumers are hesitant to buy from a wide range of brands, the more well-known green agriculture products brands are more likely to motivate consumers to buy.

Based on the above analysis, the following hypotheses were proposed.

**Hypothesis H2a:** The agribusiness image of green agriculture products brand has a positive effect on customer perceived value.
**Hypothesis H2b:** The image of agricultural products of green agriculture products brand has a positive effect on customer perceived value.
**Hypothesis H2c:** The social image of green agriculture products brands has a positive effect on customer perceived value.
**Hypothesis H2d:** The consumer image of green agriculture products brand has a positive effect on customer perceived value

#### Relationship between customer perceived value and consumption intention and the mediating role

Many studies have confirmed the link between customer-perceived value and consumption intention. Researchers have reported that consumption intention is an important outcome of brand-perceived quality value [54, 55]. A growing number of consumers are aware that their purchases directly affect many ecological issues, so many are willing to pay a premium for green products. From an empirical study of Indian consumers’ consumption behavior, it was shown that the higher the perceived value for money provided by green products, the more likely they are to pay the green price premium resulting from the incremental cost of adopting green technology mechanisms [56]. Zeithaml suggests that the price of a product or service as perceived by the consumer will have an impact on the perceived product or service to some extent. If consumers perceive that the benefits they receive are higher than the costs they have to pay, this will increase their perceived value and, consequently, their intention to consume the product [57]. Through an empirical analysis of green consumer behavior, it was found that green information, such as plastic recycling, affects consumer attitudes and customers perceived value of products, which in turn affects consumer purchase intentions [58]. Eggert’s study concluded that consumer satisfaction is one of the reasons for consumers’ purchase intention, but customer perceived value is the main motivation for consumers’ final purchase behavior. In summary, it is argued that when green agriculture product brand image and customer perceived value are compatible, it will positively influence consumers’ consumption intention, so the following hypothesis is proposed.

**Hypothesis H3:** Customer perceived value has a positive influence on consumption intention.
  1. Regarding the relationship between brand image and consumption intention, related scholars at home and abroad have done certain research. However, it has not been sufficiently proven that the brand image is dimensionalized according to the characteristics of green agriculture products to explore its influence on consumption intention, and few studies have introduced the customer perceived value variable. When companies advertise their products, their brand advantages enhance consumers’ perceptions of their products. When the consumer perception becomes stronger, the consumption intention will increase as well. In this regard, the following hypotheses are proposed:
**Hypothesis H4a:** Customer perceived value plays a mediating role in the image of agribusiness and consumption intention.
**Hypothesis H4b:** Customer perceived value plays a mediating role in the image of agricultural products and consumption intention.
**Hypothesis H4c:** Customer perceived value plays a mediating role in the image of agribusiness social and consumption intention.
**Hypothesis H4d:** Customer perceived value plays a mediating role in the image of consumer image and consumption intention.

**Fig 1:**
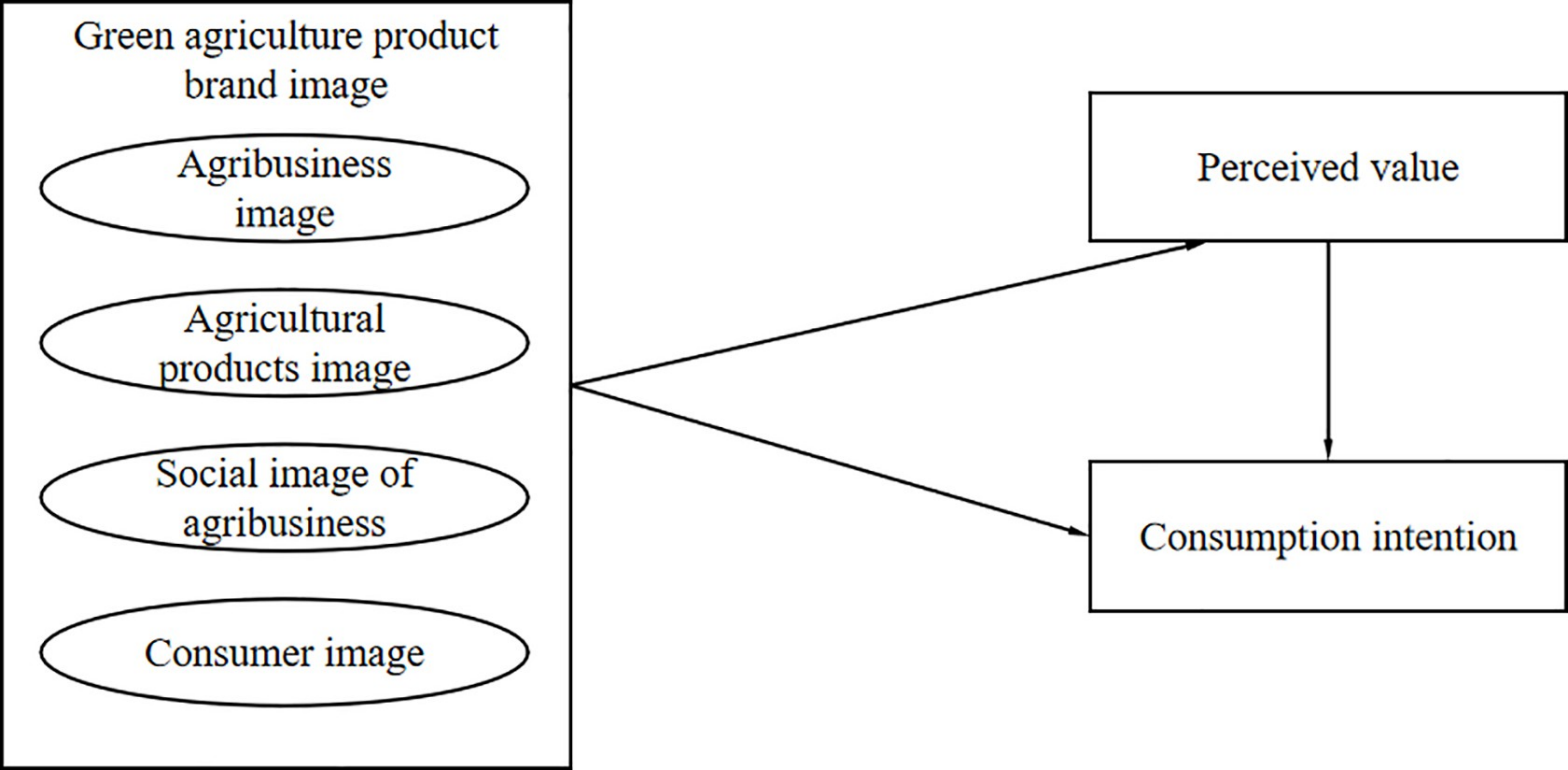
Theoretical model of this study.

Based on the above analysis, the theoretical model of green agriculture product brand image on consumption intention established in this paper is shown in Fig 1.

## Study design

### Questionnaire design and variable measurement

The questionnaires were distributed online and offline and scored on a 5-point Likert scale, with the numbers 1–5 representing the level of agreement with the questions asked. That is, 1 means "disagree," and 5 means "agree". The greater the number, the higher the level of agreement. The study was conducted with consumers of green agriculture products from all over the country, regardless of region. The scales were selected from established scales in high-level domestic and international literature. Among them, the measurement of the green agricultural brand image refers to the Bell Brand image theory model, Aaker [15], Parasuraman [59], and the scale of agricultural brand image by domestic scholars Sun Rui et al. The scale of customer perceived value refers to Xie Qiang [60] and Sweeveeney [61]. The measurement of consumption intention was based on the scales of Chan. R.Y.K [62], Rauchov [63], and Zeithaml [64]. The measurement scales for each variable are shown in Table 1.

**Table 1:** Measurement scales for each variable.

Data source	Variables	No.	Title item
Biel [18], Aaker [15], Parasuraman [59], Sun Rui et al. [45]	Agribusiness image	A1	The enterprise is well-known in the industry.
		A2	The enterprise is in a high position in the industry
		A3	The enterprise has a good reputation in the industry.
	Agricultural product image	B1	High awareness of the brand’s green agriculture products on the market
		B2	High awareness of the safety of the brand’s green agriculture products
		B3	High-cost performance of the brand’s green agriculture products
	Social image of agribusiness	C1	The company has less negative social news
		C2	The company often conducts profit-making activities.
		C3	The company often holds public welfare activities.
	Consumer image	D1	Do you think that the brand’s green agriculture products are used mainly by highly educated people?
		D2	Do you think the brand’s green agriculture products can meet individual needs?
		D3	Do you think people with high incomes mainly use the brand’s green agriculture products?
Xie Qiang [65], Sweeveeney [66]	Perceived value	F1	Do you think this brand of green agriculture products is better for your health?
		F2	Do you think this brand of green agriculture products has more nutritional value?
		F3	Do you think this brand of green agriculture products has a higher cost performance?
		F4	Do you think that buying green agriculture products from this brand can bring you a sense of well-being?
Chan. R.Y.K [67], Rauchov [68], Zeithaml [69]	Consumption intention	E1	If I purchase agricultural products, I prefer to buy green agriculture products from this brand in the same category.
		E2	I would recommend others to buy green agriculture products from this brand.
		E3	I would like to learn about the brand’s green agriculture products information and knowledge of

### Sample and data collection

Before the official questionnaire survey, the research team distributed 50 and unintelligible meanings but also conducted reliability and validity tests on the pre-survey data, deleted the questions with Cronbach’s α value less than 0.65, and added or subtracted the questions to ensure the scientific accuracy and reliability of the questionnaire, and finally obtained the official questionnaire of the study. The research was conducted from November 2022 to January 2023. Interviews were conducted with typical green agriculture product dealers in 32 provincial capitals in China, and a questionnaire was sent to consumers via email. After collecting the questionnaires, the first round eliminated those questionnaires with all the same options or patterns, and the second round eliminated those questionnaires with less than 1 minute to fill in. A total of 500 questionnaires were distributed, and 341 questionnaires were collected, with an efficiency rate of 68%. In the study sample, the first is gender, in which 48.4% of all respondents were male, and 51.3% were female. The second is age, with the highest percentage of 18-26-year-olds (34.6%). The third is the average monthly income, of which the most number of respondents, 30.8%, had a monthly income of less than $3,000. The descriptive statistical results of the final survey samples collected are shown in Table 2. In terms of gender, there are slightly more women than men. In terms of age, older and younger consumers are the least because these people are less exposed to the concept and knowledge of green agriculture products, while young people and middle-aged people are often exposed to new things and have a stronger awareness of body protection so that they will choose green agriculture products more often. With regard to the composition of wages, it is clear that the price of green agriculture products is higher than that of ordinary agricultural products. Hence, consumers have to have a certain financial basis to choose green agriculture products.

**Table 2:** The basic information of survey sample.

Statistical Variables		Sample Size	Proportion (%)
Gender	Male	165	48.4%
	Female	175	51.3%
Age	≤18	9	2.6%
	18–26	118	34.6%
	27–35	50	14.7%
	36–44	91	26.7%
	≥45	73	21.4%
Education background	Junior high school and below	22	6.5%
	High school	38	11.1%
	Junior college	60	17.6%
	Undergraduate	137	40.2%
	Master’s degree or above	84	24.6%
Monthly income	≤3000 RMB	105	30.8%
	3000-5000RMB	73	21.4%
	5000-8000RMB	80	23.5%
	8000-10000RMB	32	9.4%
	≥10000RMB	51	15%

## Model test results

### Scale reliability and validity tests

In this study, a total of 6 variables were used in the formal questionnaire, with 3 to 4 measures under each variable, for a total of 19 measures. The reliability of the questionnaire was analyzed with the help of SPSS26.0 software and by calculating the reliability (CR) and the average variance extracted (AVE) through the factor loadings of the validated factor analysis. The results are shown in Table 3.

**Table 3:** The test of model reliability and validity and confirmatory factor analysis.

Latent variable	Test variables	Standardized factor loadings	Cronbach’s α	CR	AVE
Agribusiness image	A1 A2 A3	0.789 0.838 0.838	0.807	0.862	0.676
Agricultural product image	B1 B2 B3	0.809 0.799 0.797	0.779	0.844	0.643
Social Image of Agribusiness	C1 C2 C3	0.743 0.781 0.785	0.712	0.814	0.593
Consumer image	D1 D2 D3	0.795 0.800 0.737	0.743	0.821	0.605
Customer perceived value	F1 F2 F3 F4	0.809 0.738 0.728 0.823	0.849	0.858	0.602
Consumption intention	E1 E2 E3	0.893 0.896 0.836	0.934	0.908	0.766

As shown in Table 3, Cronbach’s α was greater than 0.7 for all six variables, the combined reliability (CR) was greater than 0.7, and the average variance extracted (AVE) was greater than 0.5, indicating that the scale has good internal consistency and convergent validity. The correlation coefficients and the square root of AVE were then calculated for the six variables, and the results are shown in Table 4.

**Table 4:** Results of descriptive statistics and differential validity tests.

No.	Variable	Mean value	Standard deviation	1	2	3	4	5	6
1	Agribusiness image	3.9	0.90	0.822					
2	Agricultural product image	4	0.87	0.261**	0.801				
3	Social image of agribusiness	4	0.86	0.144**	0.172**	0.770			
4	Consumer image	3.7	1	0.222**	0.278**	0.187**	0.778		
5	Perceived value	4.1	0.79	0.250**	0.277**	0.375**	0.384**	0.776	
6	Consumption intention	3.1	1.5	0.321**	0.356**	0.348**	0.343**	0.458**	0.875

Note: The diagonal number is the square root of the AVE of the factor

*** indicates P < 0.001

** indicates P < 0.010

* indicates P < 0.0

As shown in Table 4, all the variables are significantly correlated with each other, and the Spearman’s correlation coefficient show that the correlation between each dimension and consumption intention and customer perceived value is positive, which tentatively verifies the hypothesis proposed in this paper. The square root of AVE of all six variables is greater than the number of two correlations between the variables, which indicates that the questionnaire has good discriminant validity.

### Model road worthiness test

In this study, eight indicators were selected to evaluate the fit of the structural equation model constructed in this paper. The data were analyzed by SPSS26.0 software, and the results of the preliminary fitting of the structural equation are shown in Table 5.

**Table 5:** Model fit test results.

Matching index	Recommended value	Fitted value
χ^2^	The smaller, the better	337.774
χ^2^/df	<3.0	2.466
GFI	>0.9	0.905
AGFI	>0.8	0.869
RMSEA	<0.08	0.066
NNFI	>0.9	0.896
IFI	>0.9	0.935
TLI	>0.9	0.918

As can be seen from Table 5, the results of the preliminary fitting of the structural equation in this paper, except for the NNFI, which is slightly lower than the proposed value, all other indicators are within the ideal range, indicating that the structural equation model used in this study to test the hypothesis is scientific and reasonable.

As shown in Table 6, Agribusiness image, Agricultural product image, Social image of agribusiness, and Consumer image have a significant positive effect on perceived value (β = 0.147, CR = 2.267, P < 0.050; β = 0.142, CR = 2.114, P < 0.050; β = 0.313, CR = 4.497, P < 0.001; β = 0.253, CR = 3.654, P < 0.001), Hypothesis H2a, H2b, H2c, and H2d hold. Agribusiness image, agricultural product image, Social image of agribusiness, consumer image, and customer perceived value have a significant positive effect on consumption intention (β = 0.131, CR = 2.183, p < 0.050; β = 0.171, CR = 2.708, p < 0.010; β = 0.175, CR = 2.683, p < 0.010; β = 0.150, CR = 2.318, p < 0.050; β = 0.234, CR = 3.494, p < 0.001), assuming that H1a, H1b, H1c, H1d, and H1c have a significant positive effect on consumption intention (β = 0.131, CR = 2.183, p < 0.050), Hypothesis H1b, H1c, H1d, and H3 hold.

**Table 6:** Significance test results of path coefficients.

Path relationship			Standardized path factor	SE	CR	Significance P	Conclusion
Perceived Value	←	Agribusiness image	0.147	0.066	2.267	0.023	H2a hold
Perceived Value	←	Agricultural product image	0.142	0.076	2.114	0.034	H2b hold
Perceived Value	←	Social image of agribusiness	0.313	0.068	4.497	***	H2c hold
Perceived Value	←	Consumer image	0.253	0.108	3.654	***	H2d hold
Consumption intention	←	Agribusiness image	0.131	0.084	2.183	0.029	H1a hold
Consumption intention	←	Agricultural product image	0.171	0.098	2.708	0.007	H1b hold
Consumption intention	←	Social image of agribusiness	0.175	0.088	2.683	0.007	H1c hold
Consumption intention	←	Consumer image	0.150	0.139	2.318	0.020	H1d hold
Consumption intention	←	Perceived Value	0.234	0.093	3.494	***	H3 hold

### Intermediation effect test

The validation of H4a-H4d was performed by setting the Bootstrap parameter in AMOS 24.0 software to test the mediation effect of customer perceived value, and the test results are shown in Table 7.

**Table 7:** Mediating effects test based on bootstrap.

Type	Effect	Effect value	Standard error SE	Boot CI 95% confidence interval		Conclusion
				lower bound	upper bound	
Agribusiness image →Perceived value→ Consumption intention	Direct effect	0.131	0.063	0.009	0.254	H4a hold
	Indirect effect	0.034	0.023	0.002	0.097	H4a hold
Agricultural product image→ Perceived value→ Consumption intention	Direct effect	0.171	0.068	0.028	0.298	H4b hold
	Indirect effect	0.033	0.020	0.005	0.091	H4b hold
The social image of agribusiness→ Perceived value→ Consumption intention	Direct effect	0.175	0.075	0.034	0.324	H4c hold
	Indirect effect	0.073	0.032	0.026	0.163	H4c hold
Consumer image→ Perceived value→ Consumption intention	Direct effect	0.150	0.069	0.000	0.274	H4d hold
	Indirect effect	0.059	0.029	0.016	0.141	H4d hold

From Table 7, the confidence interval between the Agribusiness image-customer perceived value-consumption intention path is [0.002, 0.097], which does not contain 0, meaning that customer perceived value plays a mediating effect in Agribusiness image and consumption intention, hypothesis H4a holds. The confidence interval between Agricultural product image—customer perceived value—consumption intention paths is [0.005, 0.091], which does not contain 0, indicating that customer perceived value plays a mediating effect in Image of agricultural products and consumption intention, and hypothesis H4b holds. The confidence interval between Social image of agribusiness—customer perceived value—consumption intention paths is [0.026, 0.163], which does not contain 0, indicating that customer perceived value plays a mediating effect in Social image of agribusiness and consumption intention, and hypothesis H4c holds. The confidence interval between the Consumer image-customer perceived value-consumption intention path is [0.016, 0.141], which does not contain 0, indicating that consumer perceived value plays a mediating effect in consumer image and consumption intention, and hypothesis H4d holds.

## Research findings and management implications

### Research findings

Based on the SOR model and brand image theory, this study constructed a hypothetical model of green agricultural products brand image influencing consumers’ purchase intention with perceived value as mediators. After empirical analysis of 341 questionnaires, the following conclusions are obtained:

In this study, the green agriculture product brand image is divided into four dimensions: Agribusiness image, Agricultural product image, the social image of agricultural enterprises, and Consumer image. Through the empirical analysis, Agribusiness image, Agricultural product image, Social image of agribusiness, and Consumer image all have a significant positive effect on consumption intention. It can be illustrated that when a company raises its visibility, strengthens its corporate culture in society, enhances its position in the industry it belongs to, improves its reputation in society, provides some convenient services to consumers, often makes concessions, and deals with some bad opinions of consumers in a timely manner, it will convey a good corporate image to consumers, which in turn will enhance consumers’ favorable feelings towards the company and thus generate consumption intention.

In this study, customer perceived value is used as a mediating variable between green agriculture product brand image and consumption intention to investigate its mediating role between green agriculture product brand image. Through empirical analysis, consumer customer perceived value plays the mediating role between green agriculture product brand image and consumption intention. According to Zeithaml [58], the customer perceived value is divided into two important factors: the perceived benefit and the cost to the customer. In this study, we consider a single dimension of customer perceived value, including price, taste, nutrition and health, and economic benefits of the produce. It can be shown that when green agriculture products have good cost performance, nutrition, and health, taste or can bring satisfaction and happiness to consumers, it will enhance the customer perceived value of the brand green agriculture products. Consumers will increase their consumption intention when they have a better-perceived value of green agriculture products.

### Theoretical contributions

This paper constructs a theoretical model of green agriculture product brand image on consumption intention. Previous studies have considered customer-perceived value as an important factor influencing consumption intention. The impact of different dimensions of customer perceived value on consumption intention is studied, or brand image is used as a single mediating variable to study the impact on consumption intention. Fewer studies have examined the impact of brand image on consumption intention as a major influencing factor and divided it into different dimensions. In this paper, based on SOR Model, green agriculture product brand image is divided into four dimensions: Agribusiness image, Agricultural product image, Social image of agribusiness, and Consumer image. It also introduces customer perceived value as a mediating variable to explore the influence mechanism and mediating effect on the consumption intention of green agriculture products, which enriches the brand image theory, customer perceived value theory, and their related studies. The study also introduces customer perceived value as a mediating variable to explore the influence mechanism and mediating effect on the consumption intention of green agriculture products.

### Managerial contributions

From the research hypothesis verification, it can be seen that shaping a good brand image is an important factor for the success of agricultural products enterprises. Brand image enhancement can increase the intangible assets of the brand, improve the competitiveness of the brand, and then improve the green agricultural products consumption intention to help the green sustainable development of agriculture. For this reason, this study proposes the following management suggestions.

First, enterprises should strengthen the awareness of creating a brand image. Green agriculture product brand image can be built from the four dimensions of the image to enhance and shape, which is most important to enhance the shaping of the Image of agricultural products and Social image of agribusiness. Enterprises should update and upgrade their brand value while completing basic tasks such as processing agricultural products, promoting and marketing, and actively holding public welfare activities. Enterprises can establish a quality traceability platform for organic products and green agriculture products and build a whole-process traceability system from the field to the table to ensure the quality of products and improve consumer brand loyalty to help sustainable consumption.

Secondly, there are more brands of green agriculture products on the market. Still, due to the lack of comprehensive and systematic management in the promotion of enterprises, the influence of the relevant brands in the market has not been given full play. The COVID-19 epidemic at the end of 2019 has caused a huge impact on traditional offline business entities, signaling that future business entities can no longer rely entirely on offline physical store marketing but should make full use of the advantages of online promotion. Therefore, enterprises should establish an Internet communication mindset to enrich green agriculture products promotion and marketing channels and advertise their products and culture through WeChat and other self-media platforms for precise advertising to different target groups. Only by combining both online and offline channels can we maximize the effect of communicating the brand image of green agriculture products. In order to prevent false propaganda, green agriculture product companies should set up professional marketing teams and adopt advanced operation methods for online businesses. At the same time, the quality of the products must be guaranteed when marketing online because it is not only about the reputation of the company’s brand but also can destroy the trust of consumers in online marketing.

Third, along with the improvement of people’s living standards, people not only have higher requirements for the quality of agricultural products but also gradually increase the external requirements for the packaging of agricultural products. Therefore, in order to meet the spiritual and cultural needs of consumers, the design of green agriculture product brand image must keep pace with the times and be innovative. Enterprises and governments should pay attention to consumers’ emotional preferences for green agriculture product brand image and can obtain the needs of different consumer groups by means of research, etc. For example, young people may prefer unique packaging design, while middle-aged people are more concerned about the nutritional value of agricultural products, etc. Hence, companies can design personalized packaging for different consumer groups to attract consumers’ attention. At the same time, it can also integrate more elements of local customs in the packaging design of green agriculture products to convey the local brand concept to consumers to reflect the brand personality so that consumers get emotional recognition and thus improve the brand competitiveness and realize the cultural value into economic value.

## Limitations of the study and future research

There are three main limitations and future research directions of this study. First, this study conducted a random sample survey in the form of questionnaire, especially in the form of online distribution. As some interviewees had a certain understanding of the research topic, the conclusions are easily inconsistent with the real thoughts of these interviewees. Future studies could consider using scientific and technical means to improve the accuracy of data recovery, or mathematical analysis and experimental methods to overcome the limitations of research methods. Second, the study was conducted with Chinese consumers, and the data was sourced from China. This may be a limitation of the study because Chinese culture is different from that of other countries, and Chinese consumers do not have the same awareness of green agriculture products as consumers in other countries. Future research should extend the scope of the study to other countries outside of China as much as possible. Thirdly, the research on the dimensions of green agriculture product brand image is very little at present, and it has been in the ambiguity and has not been unified. The dimensions of the brand image of agricultural products in this study are also based on the research on the dimensions of the brand image at home and abroad and the research of a few scholars on the dimensions of the brand image of agricultural products in China, and the dimensions of the brand image of green agriculture products are established, which inevitably have some shortcomings. In the future, research on green agriculture product brand image can be combined with various fields (sociology, psychology, medicine, etc.) to make the study of green agriculture product brand image more extensive and relevant to the times.

## Supporting information

S1 File Data. (CSV)
